# Effects of Youth Flexible Assertive Community Treatment: outcomes of an 18-month observational study

**DOI:** 10.1007/s00127-023-02508-x

**Published:** 2023-06-06

**Authors:** Marieke Broersen, Daan H. M. Creemers, Nynke Frieswijk, Ad A. Vermulst, Hans Kroon

**Affiliations:** 1 GGZ Oost Brabant, Oss, The Netherlands; 2 Tranzo-Tilburg School of Social and Behavioral Sciences, Tilburg University, Tilburg, The Netherlands; 3 Accare, Groningen, The Netherlands; 4 Trimbos Institute, Utrecht, The Netherlands

**Keywords:** Mental health services, Adolescent Mental Health, Flexible Assertive Community Treatment, Integrated care approach, Early intervention, Assertive outreach

## Abstract

**Purpose:**

This Multicenter Youth Flexible ACT Study examined the effect of Youth Flexible Assertive Community Treatment on symptomatic, social, and personal recovery outcomes of adolescents dealing with multifaceted psychiatric and social care needs who do not readily engage in regular office-based mental health services.

**Methods:**

Newly admitted clients (*n* = 199) aged 12–24 years from 16 Youth Flexible ACT teams participated in this observational prospective cohort study. Client and practitioner questionnaires were administered every 6 months, up to 18 months. Latent growth curve analyses were conducted to examine changes in symptomatic, social, and personal recovery outcomes throughout Flexible ACT.

**Results:**

Our analyses of client-reported outcomes showed a decrease in overall psychosocial difficulties, depressive symptoms, and subclinical psychosis symptoms. Moreover, outcomes showed improved social interaction with peers, quality of life, and feelings of empowerment and fewer contacts with the police/legal system. In addition, analyses of clinician-reported outcomes showed a decrease in problems related to family life, peer relationships, school/work attendance, emotional symptoms, and attentional problems. Problems related to personal finance, school and work status, substance misuse, disruptive and aggressive behavior, self-injury, and self-care and independence remained unchanged.

**Conclusion:**

Our results showed that clients participating in Youth Flexible ACT improved in symptomatic, social, and personal recovery outcomes over 18 months. With its integrated approach and personalized care, this service delivery model is promising for adolescents unable to engage successfully in regular (office based) mental health support services.

**Supplementary Information:**

The online version contains supplementary material available at 10.1007/s00127-023-02508-x.

## Introduction

As the demand for mental health treatment among young people increases, barriers to accessing and engaging in mental healthcare services are becoming more visible. Systematic inadequacies in the provision of mental healthcare include long wait times, difficulty accessing (appropriate) treatment, insufficient cooperation and communication between organizations, and discontinuity of care during the transition from Child and Adolescent Mental Health Services (CAMHS) to Adult Mental Health Services (AMHS) [1–4]. Together, these issues lead to the inability to reach or engage young people in care.

Adolescents struggling with interrelated psychiatric and social problems are particularly vulnerable to the abovementioned service design deficiencies. This subgroup of young people experiences multiple difficulties in everyday life, such as problems with educational and vocational attendance, peer relationships, housing, the legal system, and/or personal finance [5–8]. Because of the multifaceted care needs required to address these problems, various professionals from different institutions need to be involved. In addition, many of these adolescents have often already had dissatisfactory experiences with mental health services, which leads to decreased trust in the services. In sum, these adolescents find it difficult to engage in traditional office-based mental health services due to the barriers and limitations of the care system [4, 9–11]. 

Several novel integrated youth-friendly care approaches have been developed to tackle the aforementioned service design issues, such as ACCESS Open Minds in Canada and Jigsaw in Ireland [12–15]. Yet, such initiatives do not specifically address the developmental needs of young people with more severe and enduring mental health presentations. Exceptions are the “early intervention psychosis” teams and the AMYOS (Assertive Mobile Youth Outreach Service) model [16]. In the Netherlands, Youth Flexible Assertive Community Treatment (Flexible ACT) teams have been set up to provide long-term integrated outreach care specifically for young people (up to 24 years of age) that (1) have wide-ranging and interrelated (persistent and enduring) psychiatric and social care needs and (2) do not readily engage in regular office-based mental health services. The multidisciplinary teams work closely with the adolescents, their families, and/or other key support figures, address their age-related developmental needs, and support them in their personal, social, and symptomatic recovery. Since 2014, Youth Flexible ACT teams have been deployed widely throughout the Netherlands. Nowadays, around 80 teams are active or under development [17]. 

Although Youth Flexible ACT is widespread in the Netherlands and gaining international appraisal, with the first teams being implemented in Norway, only a limited body of research has examined the effects of this integrated service delivery model. Two Dutch pilot studies [6, 18] showed preliminary evidence of a reduction in behavioral problems, family life problems, hallucinations and delusions, attentional problems, emotional symptoms, self-injury, and peer problems. Indications for the effectiveness of Youth Flexible ACT may also be derived from the broader domain of Youth Assertive Community Treatment (Youth ACT) programs. A systematic review of 13 studies revealed that Youth ACT supports improved mental health and general functioning [19]. Other recently published studies have shown encouraging clinical recovery rates in adolescents cared for by ACT teams [16, 20]. In contrast to regular ACT, Flexible ACT can provide dynamic levels of care and encompass a multi-agency approach delivering psychiatric treatment and social support for wide-ranging problems. In short, studies into youth-integrated outreach models show promising treatment outcomes. Yet, evidence regarding outcomes supporting the effectiveness of the Youth Flexible ACT model remains slim. 

With our Youth Flexible ACT Multicenter Study, we aimed to investigate the effects of this Dutch client-centered service delivery model and contribute to developing accessible and integrated mental healthcare programs for adolescents with persistent and multiple care needs who find it difficult to engage in traditional mental health services. In this article, we aimed to examine change in symptomatic, social, and personal recovery outcomes throughout Youth Flexible ACT. Based on the extant literature, we hypothesized that adolescents receiving Youth Flexible ACT would show a reduction in the severity of mental health difficulties and improved social functioning over the 18-month care period. 

## Method

### Study design

The Multicenter Youth Flexible ACT Study is a longitudinal observational prospective cohort study of 16 Youth Flexible ACT teams from seven mental healthcare institutions throughout the Netherlands. Adolescents and their mental health workers were asked to complete a baseline and 3 follow-up measurements every 6 months, totaling four measurements (*T*0, *T*1, *T*2, *T*3; data collection period October 2016–January 2020). See our Study Protocol for an extensive description of the research setting, data collection, and procedure [21].

### Study inclusion criteria

The study participants comprised 12–24 years old adolescents who received Youth Flexible ACT at one of the participating mental healthcare organizations. Young people are eligible for Youth Flexible ACT if they: (1) are < 24 years of age; (2) are diagnosed with a mental health disorder (or presumptive diagnosis); (3) experience difficulties in multiple areas of daily life; (4) are not able to attend office-based treatment due to complexity of mental illness or actively refuse contact; (5) face family system problems and/or parenting issues; (6) live in the district of the Youth Flexible ACT team. Additionally, the following research inclusion criteria were used: clients had to be between 12 and 24 years of age, have sufficient knowledge of the Dutch language, and provide written informed consent (along with parent/caregivers’ consent).

### Youth Flexible ACT

The Youth Flexible ACT model is an adapted variant of the existing version of the Adult Flexible ACT, which is the standard service delivery model for people with severe mental illness in the Netherlands [22, 23]. Flexible ACT teams incorporate an integral focused care approach, providing care across several domains, including psychiatric, addiction, and supportive care. The teams are composed of employees from different organizations (multi-agency approach) and collaborate closely with professionals from (other) care organizations (e.g., AMHS, CAMHS, addiction treatment services, intellectual disability services, and community social services). Flexible ACT aims to enhance continuity of care by delivering and coordinating psychiatric treatment and practical support by the same team of professionals, as well as adjusting care to each client’s needs (through individual case management and intensive team care). Unlike Adult Flexible ACT, Youth Flexible ACT includes a systemic family therapist, an employment and education specialist, and parent and family counselors. The teams stimulate children, adolescents, and young adults in the aspects of personal identity, social contacts, school, work, and leisure. They boost their resilience by developing life skills appropriate to their life stages and transitions. Youth Flexible ACT provides recovery and development-oriented interventions addressing three recovery domains [24]: symptomatic (minimize clinical symptoms), social (i.e., regaining everyday functioning in education, work and leisure, social relationships, and self-care and living) and personal (i.e., regaining a grip on their life, establishing a positive identity, living a meaningful life) [25]. Furthermore, the teams are expected to provide high-quality care according to the Flexible ACT model guidelines, including clinical practice guidelines and evidence-based practices. The Centre for Certification and Flexible ACT (CCAF) determines model adherence via audits [17]. Our case study provides a detailed description of Youth Flexible ACT [26].

### Data collection procedure

Youth Flexible ACT team members asked adolescents to participate in the study during their regular care intake process. After signing informed consent, participants and the mental health workers were asked to complete a baseline measurement. The participants completed the questionnaires during their regular appointment with a familiar mental health worker or independently in their own time. Researchers were in close contact with mental health workers and informed them timely on (1) completing the follow-up questionnaires (*T*1, *T*2, *T*3) about their enrolled clients and (2) notifying their enrolled clients to complete their follow-up questionnaires. Both paper and online questionnaires were available, although online versions were preferred to minimize the chance of missing data. An online data system was used to collect the data. Confidentiality of the data was guaranteed through a two-factor authentication login procedure. Adolescent participants received a remuneration of €10,- per assessment. Trimbos Ethics Committee approved this study and its procedures (201607_75-FACT2).

### Study sample

During the enrolment period (October 2016–June 2018), 199 eligible clients signed the informed consent to participate and completed baseline questionnaires. This client group (69% was 18 years of age or older) showed a high diversity of severe psychiatric and social problems associated with significant trauma and developmental, mood, and anxiety disorders (Table 1) [8]. Their development in multiple life domains was hindered, especially since one-third did not attend a school or have a job, and almost all adolescents showed problems with family life and peer relationships. About half reported experiencing poor quality of life. Other frequently reported difficulties were substance misuse problems, the involvement of the police/legal system, problems with intellectual functioning, and personal finance. Before the Youth Flexible ACT referral, most adolescents had been involved with office-based (specialized) mental healthcare (see our baseline paper for a detailed description of the Youth Flexible ACT client group) [8].

**Table 1:** Sociodemographic and clinical characteristics of study sample

	*M*	*SD*
Age (*n* = 199)	18.57	2.49

	*n*	%
Age: 15 to 22 years of age	175	87.9
Age: ≥ 18 years	137	69
Girls	101	50.8
Born in the Netherlands	189	95
Having school and/or employment	132	66.3
Mental health disorders		
Anxiety and mood disorders	90	45.2
Trauma and stressor related disorders	54	27.1
Autism spectrum disorder	52	26.1
Attention-deficit/hyperactivity disorder	43	21.6
Personality disorder	31	15.6
Disruptive, impulse-control and conduct disorders	16	8
Substance use disorders	15	7.5
Psychotic disorders	7	3.5
(suspected) Below-average intellectual functioning	37	18.6
Overall functioning		
School or work-related problems	132	66.3
Financial problems	43	21.6
Involvement of the police/legal system	46	23.1
Problems with family life and relationships	166	83.4
Problems with peer relationships	149	74.8
Referral from specialized mental health care	91	45.8
Received specialized mental health care < 6 months before Youth Flexible ACT care	93	46.3
Family member receiving Flexible ACT	23	11.6

Mental health disorders are described according to the Diagnostic and Statistical Manual of Mental Disorders, 5th edition (DSM-5). The adolescents completed self-developed multiple-choice questions about going to school or having work, having financial problems, being involved with the police/legal system, and receiving care before Flexible ACT. Problems with family life and peer relationships were assessed with the HoNOSCA questionnaire (Health of the National Outcome Scales for Children and Adolescents), as reported by mental health workers. Mental health workers also provided information about the referral and family members receiving Flexible ACT

### Instruments

Table 2 presents an overview of outcomes and respective measures. Additional information about the questionnaires is described in our study protocol [21].

**Table 2:** Overview of outcomes and respective measures

Variable	Instruments			Score range	Psychometric properties
	Symptomatic domain	Social domain	Personal domain		
Client reported outcomes					
Psychosocial well-being	SDQ [27]			The total difficulties score ranges from 0 to 40, with higher scores representing more severe psychosocial difficulties The impact score (measuring the impact of the total difficulties on daily life in different domains) ranges from 0 to 10. Higher scores represent more distress and impairment in daily life	The SDQ was shown to have adequate psychometric properties, with Cronbach’s alpha coefficients of ≥ 0.70 for the SDQ total and impact score [27–29]. In our sample, Cronbach’s alpha was 0.78 for the SDQ total score and 0.74 for the impact score
Depressive symptoms	CDI-2 [30, 31]			The total score ranges from 0 to 56, with higher scores indicating more severe depressive symptoms	The CDI-2 was shown to have adequate psychometric properties, with a Cronbach’s alpha of 0.90 [30]. In our sample Cronbach’s alpha was 0.90
Psychosis risk screening	PQ-16 [32]			The total score ranges from 0 to 16, with higher scores indicating the presence of more subclinical psychosis symptoms. The distress score ranges from 0 to 18, and higher scores represent more severe distress	The PQ-16 was shown to have adequate psychometric properties, with a Cronbach’s alpha ranging from 0.77 to 0.84 for the total score [32, 33]. In our sample Cronbach's alpha was 0.82
Health-related quality of life			Kidscreen-10 [34]	Total scores are converted into *T* values (0–100), with higher scores indicating a higher health-related quality of life	The Kidscreen-10 was shown to have adequate psychometric properties, with a Cronbach’s alpha of 0.81 [34, 35]. In our sample Cronbach’s alpha was 0.82
Social support and peers		Scale ‘social support and peers’ from the Kidscreen-52 [36]		Total scores are converted into *T* values (0–100), with higher values indicating higher quality of social interaction with peers	The subscale ‘social support and peers’ was shown to have a Cronbach’s alpha of 0.84 [37] In our sample Cronbach’s alpha was 0.90
Empowerment			Subscale ‘interactional empowerment’ from the youth empowerment questionnaire (EMPO 3.1) [38]	Total scores are converted into *T* values (0–100), with higher values indicating experiencing a higher level of empowerment	The subscale ‘interactional empowerment’ was shown to have a Cronbach’s alpha of 0.79 [38]. In our sample Cronbach’s alpha was 0.76
Treatment satisfaction			On a scale of 1–10, what score would you give to the Youth Flexible ACT care you receive?	The score ranges from 1 (very bad) to 10 (very good)	
School and/or work situation		Going to school and/or having work		0 = no; 1 = yes	
Financial situation		Having financial difficulties or debts		0 = no; 1 = yes	
Involvement of the police/legal system		Having been in contact with the police/legal system in the past 6 months		0 = no; 1 = yes	
Clinician-reported outcomes					
Mental health and daily functioning	HoNOSCA items [39]: - Problems with disruptive, antisocial, or aggressive behavior - Problems with overactivity, attention, or concentration - Problems associated with hallucinations, delusions, or abnormal perceptions - Problems with emotional and related symptoms - Problems with alcohol, substance/solvent misuse - Non-accidental self-injury	HoNOSCA items [39]: - Problems with peer relationships - Problems with family life and relationships - Poor school and/or work attendance - Problems with self-care and independence		Item scores range from 0 (no problem) to 4 (severe to very severe problem) The total score is calculated as the sum of all 13 items, ranging from 0 to 52 (higher scores represent greater dysfunction severity)	The HoNOSCA was shown to have adequate psychometric properties, with a test–retest reliability coefficient of 0.69 and inter-rater reliability coefficient of 0.81 [40, 41]. In our sample Cronbach’s alpha was 0.67
Psychiatric hospital admissions		- Was your client admitted to an inpatient unit (e.g., mental health, forensic care, addiction care) in the past 6 months?		0 = no; 1 = yes	

*SDQ* The Strengths and Difficulties Questionnaire, *CDI-2* Child Depression Inventory, *PQ-16* The Prodromal Questionnaire, *EMPO 3.1* Youth Empowerment Questionnaire, *HoNOSCA* Health of the National Outcome Scales for Children and Adolescents

### Statistical analyses

Originally, we opted for an intention-to-treat analysis and created a link to the remaining questionnaires we sent via email to participants who exited Youth Flexible ACT within the data collection period. However, following up with clients after exiting Youth Flexible ACT was unsuccessful. Only a very small group (*n* = 12) did respond and had incomplete data. The analysis was, therefore, restricted to a per-protocol analysis of data from the period in which clients were enrolled in Youth Flexible ACT.

To test the hypothesis that treatment with Youth Flexible ACT is associated with improved symptomatic and functional outcomes, we applied latent growth curve analyses (LGCA) [42] using the statistical package Mplus, version 7.2 [43]. A linear growth curve between time (in months) and outcomes was assumed for each individual with intercept *i* (starting value) and slope *s* (increase or decrease per month) as parameter estimates. For the continuous outcomes, normal linear regression was used to estimate the means of the growth parameters *i* and *s*. A one-unit change in time (1 month) means that the outcome variable changes with the value of *s*. For binary and ordinal outcomes, logistic regression was used. The relationship between time and the outcome variable’s logit (log odds) was linear. The intercept *i* was fixed at zero (for model identification purposes) and the slope *s* was estimated. A one-unit change in time (1 month) means that the outcome variable’s logit (or log odds) changes with the value of *s*. Because clients were nested within 16 teams, COMPLEX was used to correct for possible non-independence of the data to obtain unbiased standard errors of the growth curve parameter estimates. To handle missingness in the data, we assumed that the missing data mechanism was Missing At Random (MAR); see also the Statistical Appendix (Online Resource, S1 Statistical Appendix). The Full Information Maximum Likelihood estimator (FIML; using all available information in the data) was used to handle missing values [44].

In our dataset, the actual time points for completing questionnaires varied. Consequently, the growth models could not be estimated with equidistant time points but with individually varying time points. Model fit indices for this kind of random growth models could not be calculated. Moreover, in longitudinal analysis, the effect of missingness on the results is related to study dropout, not intermittent missing values [45]. For intermittent missing values (e.g., respondents did not drop out of the study but did not fill out the questionnaire at one or more time points), random missingness was assumed, and it had no effect on the point estimates of *i* and *s*, but may have affected the standard errors of these point estimates (leading to higher standard errors and therefore lowering the probability of significant results). To account for study dropout, we performed sensitivity analyses (Online Resource, S1 Statistical Appendix) in which we compared the results of LGCA assuming MAR with LGCA under several conditions of Missing Not At Random (MNAR).

Effect sizes were estimated as Cohen’s *d* [46]: *d* = (*s* * duration)/*SD* with *s* as the slope (change per month) and duration as the treatment period. For *SD*, the estimated standard deviation at baseline was used (the most reliable estimate due to the absence of dropouts) for continuous outcomes. For binary or ordinal outcomes, the *SD* of the standard logistic distribution (*π*/√3=1.814) was used [47, 48]. Effect sizes of non-significant slopes were also non-significant.

## Results

### Study dropout and response rates

#### Research participants 

**Fig. 1:**
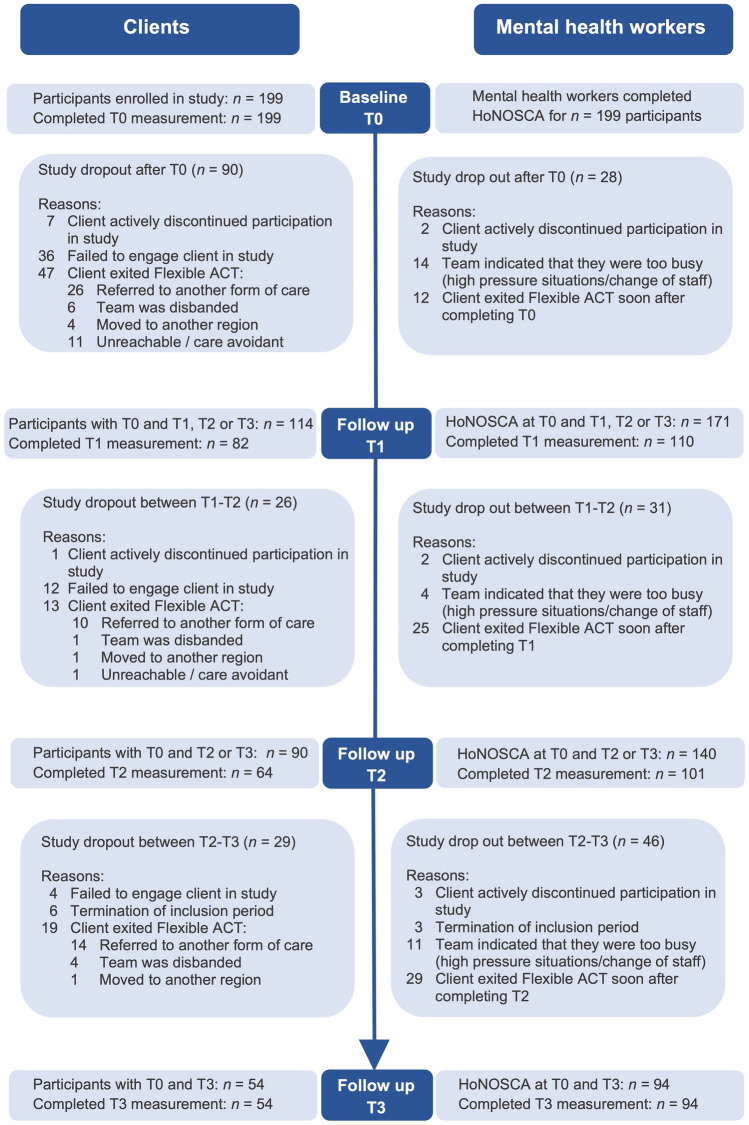
Flow diagram of study participants

Regarding study dropout, 109 (54.8%) of the 199 participants were still enrolled in the study at *T*1, 83 (41.7%) at *T*2, and 54 (27.1%) at *T*3 (Fig. 1). The most frequent reasons for study dropout were: (1) research participants exited Youth Flexible ACT and subsequently discontinued participation in the study (*n* = 79) and (2) failure to engage research participants in the study (*n* = 52). In these cases, teams indicated that they were too busy to administer the questionnaires (due to high-pressure situations, such as clients in crisis or changes in staff). Completing a research questionnaire (and to continue with the study) was not a priority if a client was in the ‘meddling’ care phase.

Not all enrolled clients completed the questionnaires at each measurement (Fig. 1). At *T*0, all 199 clients filled out the questionnaires. At *T*1, 82 of the 109 clients; at *T*2, 64 of the 83 clients; and at *T*3, all 54 clients completed the survey. The missed measurements of the enrolled clients are denoted as ‘intermittently missing’. As described above, the measurements were missing mainly at staff and organizational levels, and in other cases, clients indicated no motivation to complete questionnaires at that moment.

To get insights into the effect of study dropout, we tested whether the 145 study dropouts differed from the 54 study completers in age, gender, and symptomatic and functional outcomes completed at baseline. For the continuous variables, we used *t*-tests for independent samples; for the binary variables, we applied Chi-square tests. We found four significant results: study dropouts scored significantly lower on total difficulties (SDQ; *t* = − 2.06, *p* = 0.033), lower on SDQ impact (SDQ; *t* = − 2.71, *p* = 0.007), lower on depressive symptoms (CDI-2; *t* = − 2.31, *p* = 0.022), and lower on treatment satisfaction (*t* = − 2.01, *p* = 0.046).

#### Mental health workers 

Mental health workers completed the HoNOSCA for all 199 clients at *T*0, 110 out of 171 clients at *T*1, 101 out of 140 clients at *T*2, and all 94 clients at *T*3. We compared the total number of study dropouts (*n* = 105) with the study completers (*n* = 94) (Fig. 1). For age and the HoNOSCA total score, the *t*-test for independent samples was used, and for the 13 HoNOSCA-items (ordinal variables), the Mann–Whitney test for independent samples. No significant differences were found for these 15 variables.

### Time of response

Most adolescents completed *T*0 within 3 months (74.9%; mean 2.7 months; *SD* 2.28; range 1–14) after the start of the Flexible ACT care. *T*1 was completed after an average of 7.6 months after *T*0 (*SD* 1.53; range 5–13 months). *T*2 was completed after an average of 7.8 months after *T*1 (*SD* 2.18; range 5–16 months), and *T*3 was completed after an average of 6.7 months (*SD* 2.53; range 3–12 months) after *T*2.

Most mental health workers (70.4%; mean 3.0 months; *SD* 2.38; range 1–11) completed *T*0 within 3.0 months after the Flexible ACT care. *T*1 was completed after an average of 7.1 months (*SD* 1.92; range 2–13 months) after *T*0, *T*2 was completed after an average of 8.4 months after *T*1 (*SD* 2.97; range 4–17 months), and *T*3 was completed after an average of 7.6 months (*SD* 3.35; range 3–17 months) after *T*2.

### Latent growth curve analyses

#### LGCA for the client-reported outcomes under the assumption of MAR with varying time points and correction for team effects 

Table 3 displays means and standard deviations and the results of LGCA for the client-reported outcomes. The continuous outcome variables showed significant reductions in problems over time, all with small effect sizes. This included significant decreases (negative slopes) for the SDQ total score (psychosocial difficulties), SDQ impact score (impact of difficulties on daily life), CDI-2 total score (depressive symptoms), PQ-16 total score, and distress score (subclinical psychosis symptoms). Significant increases (positive slopes) were found for Kidscreen-10 (quality of life), EMPO 3.1 (empowerment), and Social Support and Peers (subscale Kidscreen-27). The binary outcome variables showed a negative slope for contact with the ‘Police/Legal System’, with a very large effect size. Reductions in problems were not significant for ‘School and/or work situation’, ‘Financial problems’, and ‘Satisfaction with Youth Flexible ACT’.

**Table 3:** LGCA results of client-reported outcomes

	*T*0	*T*1	*T*2	*T*3	Intercept	Slope	*p*	Cohen’s *d* effect size
	*M* (*SD*)	*M* (*SD*)	*M* (*SD*)	*M* (*SD*)				
SDQ								
Total score	16.30 (6.14)	14.88 (5.76)	14.91 (6.12)	15.18 (5.53)	16.095	− 0.061	0.035	0.22
Impact score	3.69 (2.72)	2.56 (2.15)	3.21 (2.74)	2.69 (2.46)	3.506	− 0.043	0.022	0.35
CDI-2	18.89 (10.14)	16.78 (9.35)	15.92 (9.25)	14.50 (8.41)	18.867	− 0.209	0.001	0.45
PQ-16								
Total score	5.61 (3.77)	5.48 (3.92)	5.02 (3.84)	4.67 (3.57)	5.657	− 0.043	0.031	0.25
Distress score	8.66 (7.02)	7.49 (7.20)	6.40 (6.57)	6.80 (7.10)	8.621	− 0.122	0.002	0.38
Social support and peers	63.63 (12.91)	64.40 (8.03)	66.50 (11.89)	67.37 (15.13)	63.558	0.173	0.011	0.29
Kidscreen-10	39.99 (4.54)	41.10 (4.06)	41.40 (4.70)	41.27 (4.47)	40.171	0.059	0.009	0.29
EMPO 3.1	46.90 (11.31)	48.85 (11.20)	50.18 (10.61)	52.66 (12.10)	46.921	0.243	.000	0.47
Financial problems (0 = no; 1 = yes)	0.23 (0.42)	0.24 (0.43)	0.26 (0.44)	0.23 (0.43)	0.000	− 0.062	0.55	0.75
Police/Legal system (0 = no; 1 = yes)	0.24 (0.43)	0.17 (0.38)	0.13 (0.34)	0.12 (0.32)	0.000	− 0.124	0.04	1.5
School and/or work situation (0 = no; 1 = yes)	0.66 (0.47)	0.70 (0.46)	0.52 (0.50)	0.52 (0.50)	0.000	− 0.03	0.427	0.36
Satisfaction with Youth Flexible ACT (1 = very bad; 10 = very good)	7.65 (1.76)	7.94 (1.62)	7.64 (1.86)	7.75 (1.90)	7.672	0.007	0.268	0.09

*SDQ* The Strengths and Difficulties Questionnaire, *CDI-2* Child Depression Inventory, *PQ-16* The Prodromal Questionnaire, *EMPO 3.1* Youth Empowerment Questionnaire, *HoNOSCA* Health of the National Outcome Scales for Children and Adolescents

#### LGCA for the clinician-reported outcomes under the assumption of MAR with varying time points and correction for team effects 

Table 4 displays means and standard deviations and the results of LGCA for the clinician-reported outcomes. Of the 13 ordinal outcome variables, we found negative slopes with small to medium effect sizes for 5 HoNOSCA variables related to ‘overactivity and attentional problems’, ‘emotional problems’, ‘peer relationship problems’, ‘problems with family life’, and ‘poor school/work attendance’. The total score of the HoNOSCA, a continuous variable, showed a negative slope, meaning that the total score decreased over time. The effect size was medium. Reductions in problems were not significant for ‘disruptive and aggressive behavior’, ‘self-injury’, ‘substance misuse’, ‘problems with scholastic or language skills’, ‘physical illness or disability problems’, ‘hallucinations and delusions’, ‘non-organic somatic problems’ and ‘self-care and independence’. Yet, it should be noted that roughly 30% of the clients who scored problematic (severity score 2–4) on these HoNOSCA domains at baseline returned to a non-problematic score (severity score 0–1) at *T*2 or *T*3 (see Online Resource, Table S1). The number of psychiatric hospital admissions did not change significantly throughout the treatment period.

**Table 4:** LGCA results of clinician-reported outcomes

	*T*0	*T*1	*T*2	*T*3	Intercept	Slope	*p*	Cohen’s *d* effect size
	*M* (*SD*)	*M* (*SD*)	*M* (*SD*)	*M* (*SD*)				
HoNOSCA	1.32 (1.31)	1.20 (1.25)	1.16 (1.20)	1.10 (1.24)	0.00	− 0.021	0.252	0.27
1. Problems with disruptive, antisocial or aggressive behavior	1.80 (1.21)	1.62 (1.17)	1.56 (1.11)	1.36 (1.18)	0.00	− 0.045	0.004	0.57
2. Problems with overactivity, attention or concentration	0.75 (1.15)	0.67 (1.15)	0.65 (1.13)	0.64 (1.10)	0.00	− 0.017	0.563	0.22
3. Non-accidental self-injury	0.93 (1.37)	0.87 (1.33)	0.89 (1.36)	0.96 (1.32)	0.00	0.008	0.768	0.1
4. Problems with alcohol, substance/solvent misuse	1.46 (1.35)	1.13 (1.37)	1.24 (1.37)	1.06 (1.34)	0.00	− 0.041	− 0.056	0.52
5. Problems with scholastic or language skills	0.59 (1.08)	0.55 (1.00)	0.59 (1.06)	0.53 (1.05)	0.00	− 0.024	0.322	0.3
6. Physical illness or disability problems	0.66 (1.08)	0.44 (0.88)	0.39 (0.88)	0.19 (0.65)	0.00	− 0.138	0.422	1.75
7. Problems associated with hallucinations, delusions or abnormal perceptions	0.84 (1.18)	0.61 (1.09)	0.58 (1.07)	0.63 (1.05)	0.00	− 0.028	0.117	0.36
8. Problems with non-organic somatic symptoms	2.53 (1.17)	2.27 (1.25)	2.20 (1.25)	2.18 (1.11)	0.00	− 0.036	0.009	0.46
9. Problems with emotional and related symptoms	2.25 (1.18)	2.07 (1.23)	2.01 (1.32)	1.77 (1.17)	0.00	− 0.045	0.012	0.57
10. Problems with peer relationships	1.32 (1.17)	1.22 (1.01)	1.23 (1.09)	1.10 (1.11)	0.00	− 0.042	0.239	0.53
11. Problems with self-care and independence	2.59 (1.14)	2.38 (1.16)	2.03 (1.17)	2.24 (1.71)	0.00	− 0.055	0.013	0.7
12. Problems with family life and relationships	2.24 (1.71)	1.64 (1.66)	1.85 (1.68)	1.60 (1.71)	0.00	− 0.037	0.012	0.47
13. Poor school/work attendance Total score	19.25 (7.27)	16.48 (8.21)	16.46 (8.77)	14.54 (8.50)	18.88	− 0.183	0.001	0.58
Psychiatric hospital admissions	n.a	0.148	0.133	0.065	0.00	− 0.053	0.722	0.67

*LGCA* Latent growth curve analyses, *HoNOSCA* Health of the National Outcome Scales for Children and Adolescent

## Discussion

### Main findings

This study investigated changes in symptomatic, social, and personal recovery outcomes over 18 months of Youth Flexible ACT. In line with previous Dutch pilot studies on the effects of the Youth Flexible ACT service model [6, 18], adolescents improved on both the symptomatic, social, and personal recovery domains during the treatment. Regarding (1) *symptomatic recovery*, client-reported outcomes included a reduction in overall psychosocial difficulties, depressive symptoms, and subclinical psychosis symptoms. In addition, clinician-reported outcomes involved a reduction in emotional and attentional problems. Concerning (2) *social recovery*, client-reported social outcomes included improved social interaction with peers and fewer contact moments with the police/legal system. In addition, clinician-reported outcomes showed fewer problems with school and/or work attendance, family life, and peer relationships. In terms of (3) *personal recovery*, client-reported outcomes included increased feelings of empowerment and health-related quality of life. Moreover, clients indicated that their psychosocial difficulties interfered less with everyday life. 

### Vocational and educational recovery

As outlined above, mental health workers reported decreased problems with school and/or work attendance throughout Flexible ACT. This means that adolescents who attend their classes and work more responsibly, and those who stay home, feel less resistance to finding appropriate education or work. Yet, client reports showed that education and/or employment status did not significantly change. Although the overall attitude towards education and work improved, this improvement did not result in more adolescents finding appropriate education or work. A possible reason could be that the study period of 18 months was too short to detect changes in educational and employment status. The desire to seek education or work at the onset of Youth Flexible ACT is not always immediate, and there can be a considerable delay between program enrollment and its start. Another explanation could be the type of care employed to address educational and vocational recovery varied between teams. Individual Placement and Support (IPS) is the gold standard for vocational recovery and has shown positive results for young people in a similar youth target group [49, 50]. At the time of data collection, IPS was being implemented in some of the teams.

### Clinician-reported outcomes

While the LGCA of clinician reports showed a significant reduction in emotional problems, attentional problems, problems with school and/or work attendance, and problems with family life and peer relationships, it did not show reduced problems related to the other problem areas, such as substance misuse, self-injury, hallucinations and delusions, disruptive and aggressive behavior, and self-care and independence. It is interesting to note that for the outcomes that showed a significant reduction, a much larger percentage of adolescents reported ‘problematic’ at baseline (71.1% average over 5 scores; see Online Resource, Table S1) compared to outcomes that did not show a significant reduction (29.9% average over 8 scores). This suggests that only those symptoms that were severe at baseline were reduced. Focusing on adolescents who had a ‘problematic’ score on substance misuse, self-injury, hallucinations and delusions, disruptive and aggressive behavior, and self-care and independence at baseline, roughly half (48.2–67.7% see Online Resource, Table S1) of those who participated at *T*2 or *T*3 experienced a drop in score to ‘non-problematic’. In sum, we only found improvements over time for those outcomes that were severe at baseline.

It is important to note that not all interventions commenced at the start of the study period (*T*0). This can be because clients do not always have the immediate desire to commit to intervention at the onset of Youth Flexible ACT. The study period might not have been long enough to measure effects if treatment had started later during the study period, especially when there was no fourth measurement.

### Strengths and limitations

This observational cohort study had several strengths: the naturalistic character of the study, the use of both clinician and client informants, the monitoring recovery on three domains simultaneously, and the participation of multiple teams across the Netherlands. Some limitations should be considered while interpreting our results.

First, we had no control or comparison group; therefore, outcomes could have also improved if adolescents had received another type of treatment or no treatment at all. We attempted to follow-up with adolescents who exited care to allow for some comparison between adolescents in and outside Youth Flexible ACT. However, these adolescents were difficult to reach and did not respond to our queries, which means that we only have data for adolescents who received Youth Flexible ACT at the time of completing the questionnaires. Our conclusions are restricted to adolescents who received Youth Flexible ACT. 

Second, we had a high attrition rate in the follow-up. Although this is not surprising for a client group with an intensive intervention and four assessment points over 18 months, it could result in attrition bias in which participants who dropped out of the study differed from those who remained. In our study, dropouts were less satisfied with Flexible ACT than those who remained. However, dropouts still reported positive satisfaction with Flexible ACT (7.49 on a scale of 10, *SD* 1.78). In addition, the client-reported data showed that adolescents with less severe problems on SDQ (psychosocial difficulties) and CDI-2 (depressive symptoms) were more likely to drop out of the study. In addition, mental health workers did not report any differences in the HoNOSCA domains between dropouts and those who remained. As dropout data showed that most study dropouts also exited Youth Flexible ACT, it is possible that adolescents with less severe problems were less motivated to engage in treatment and participate in our study.

Moreover, it could be that these adolescents were no longer in need of Flexible ACT and a lighter form of care was more appropriate. Consequently, the follow-up measurements included more clients with severe problems on some self-reported scales. This could be an indication that the external validity of our results is limited to those adolescents who had severe problems at baseline. Additionally, the previous discussion section suggested that Youth Flexible ACT is more likely to reduce symptoms for individuals scoring higher at baseline. In sum, our results suggest that Youth Flexible ACT can be more useful for those adolescents and those domains that show severe problems at the onset.

Furthermore, the dropout data also showed that some adolescents had already exited Youth Flexible ACT between *T*0 and *T*1 (Fig. 1). Team members indicated that another form of care was more appropriate. On one hand, the referral to the Flexible ACT team might not have been right in the first place. This suggests that triage conducted at the point of entry could have been more accurate. On the other hand, these young people might have had difficulty accessing and engaging in regular mental healthcare services and possibly required a Flexible ACT approach (team’s easy accessibility, flexibility and outreaching character) to find the most appropriate form of care. Youth Flexible ACT could then act as a consultation hub for this group of clients.

Also, we performed a multitude of statistical tests, which could indicate the need for multiple test corrections. While such tests indeed decrease the probability of a type-I error (false-positive: the chance to label non-existing effects as significant), they also increase the probability of a type-II error (false-negative: the chance to miss real effects). The use of multiple test adjustments is, therefore, advised in experimental designs, especially in the setting of confirmatory clinical trials [51]. However, as the purpose of our study is more exploratory, we did not opt to perform multiple test corrections. It is, therefore, important to note that all of our reported effects should be considered as exploratory findings, and warrant confirmation in future research. 

Finally, effect sizes from non-experimental studies tend to be larger than those from experimental studies with a control group [52]. The magnitude of the effect size should, therefore, be considered in relation to our observational research design.

### Clinical implications and future research

Our study findings indicated that the Youth Flexible ACT service delivery model is promising for adolescents unable to engage successfully in regular (office-based) mental health support services. These adolescents with persistent psychiatric and social care needs are at risk of long-term problems. Even though the period of this study is relatively short (i.e., 18 months), the adolescents included already showed signs of improvement on important outcomes. Future research should include long-term follow-up (for instance, 3 years) to understand the recovery process at the symptomatic, social, and personal levels (for example, to examine how the school and/or work situation is improving) and to evaluate whether clients maintain a positive development after the treatment. Moreover, we need to explore the types of health care services provided during Flexible ACT and specific elements of the Youth Flexible ACT model that contribute to change over time to make the intervention more precise and personalized.

In summary, the Youth Flexible ACT model is an integrated and recovery- and developmental-oriented service delivery model for transitional age youth with persistent and multifaceted mental health and social care needs who find engagement in mainstream mental health services difficult. This study provides promising initial evidence of Youth Flexible ACT being associated with improved symptomatic, social, and personal recovery. Future research is needed to further corroborate the effects of this service delivery model.

### Supplementary Information

Below is the link to the electronic supplementary material.

Supplementary file1 (PDF 91 KB)

Supplementary file2 (PDF 81 KB)

## Data Availability

The datasets generated for this article are not readily available due to ethical, legal, and privacy restrictions. Requests to access the datasets should be directed to the corresponding author.

## Abbreviations

ACT: Assertive Community Treatment
AMHS: Adult Mental Health Services
AMYOS: Assertive Mobile Youth Outreach Service
CAMHS: Child and Adolescent Mental Health Services
CCAF: Centre for Certification ACT and Flexible ACT
CDI-2: Child Depression Inventory 2
DSM-5: Diagnostic and Statistical Manual of Mental Disorders, 5th edition
EMPO 3.1: Youth Empowerment Questionnaire 3.1
FIML: Full Information Maximum Likelihood estimator
Flexible ACT: Flexible Assertive Community Treatment
HoNOSCA: Health of the National Outcome Scales for Children and Adolescents
IPS: Individual Placement and Support
LGCA: Latent growth curve analyses
MAR: Missing At Random
MCAR: Missing Completely At Random
MNAR: Missing Not At Random
PQ-16: The Prodromal Questionnaire 16
SDQ: The Strengths and Difficulties Questionnaire
STROBE: Strengthening the reporting of observational studies in epidemiology
