# Supplementary material for: Effects of Youth Flexible Assertive Community Treatment: outcomes of an 18-month observational study
Source: Soc Psychiatry Psychiatr Epidemiol. 2023 Jun 6;59(5):745–58. doi: 10.1007/s00127-023-02508-x (PMC11087363; doi:10.1007/s00127-023-02508-x)
Supplement: Supplementary file 1 — Supplementary file1 (PDF 91 KB) [file 127_2023_2508_MOESM1_ESM.pdf]

Table S1 Problematic- and Non-Problematic Scores on HoNOSCA Items

	Problematic score at baseline (T0)	Follow-up (T2 or T3) of adolescents with a problematic score at T0				LGCA significance
		Missing	Problematic score	Non-problematic score	Non-problematic / Problematic ratio	
**HoNOSCA**						
1. Problems with disruptive, antisocial or aggressive behavior	N = 78 out of 199 (39.2%)	N = 27 (34.6%)	N = 24 (30.8%)	N = 27 (34.6%)	52.9%	NS
2. Problems with overactivity, attention or concentration	N = 113 out of 199 (56.8%)	N = 35 (31.0%)	N = 46 (40.7%)	N = 32 (28.3%)	41.0%	*p* = .004
3. Non-accidental self-injury	N = 43 out of 199 (21.7%)	N = 16 (37.2%)	N = 14 (32.6%)	N = 13 (30.2%)	48.2%	NS
4. Problems with alcohol, substance/solvent misuse	N = 53 out of 199 (26.6%)	N = 24 (45.3%)	N = 15 (28.3%)	N = 14 (26.4%)	48.3%	NS
5. Problems with scholastic or language skills	N = 93 out of 199 (46.7%)	N = 30 (32.3%)	N = 25 (26.9%)	N = 38 (40.9%)	60.3%	NS
6. Physical illness or disability problems	N = 34 out of 199 (17.1%)	N = 8 (23.5%)	N = 9 (26.5%)	N = 17 (50.0%)	65.4%	NS
7. Problems associated with hallucinations, delusions or abnormal perceptions	N = 40 out of 199 (20.1%)	N = 15 (37.5%)	N = 9 (22.5%)	N = 16 (40.0%)	64.0%	NS
8. Problems with non-organic somatic symptoms	N = 46 out of 199 (23.1%)	N = 15 (32.6%)	N = 10 (21.7%)	N = 21 (45.7%)	67.7%	NS
9. Problems with emotional and related symptoms	N = 161 out of 199 (80.9%)	N = 45 (28.0%)	N = 87 (54.0%)	N = 29 (18.0%)	25.0%	*p* = .009
10. Problems with peer relationships	N = 149 out of 199 (74.9%)	N = 46 (30.9%)	N = 63 (42.3%)	N = 40 (26.8%)	38.8%	*p* = .012
11. Problems with self-care and independence	N = 89 out of 199 (44.7%)	N = 32 (35.6%)	N = 28 (31.1%)	N = 30 (33.3%)	51.7%	NS
12. Problems with family life and relationships	N = 166 out of 199 (83.4%)	N = 52 (31.1%)	N = 83 (50.0%)	N = 31 (18.7%)	27.2%	*p* = .013

13. Poor school/work attendance	N = 119 out of 199 (59.8%)	N = 37 (31.1%)	N = 46 (38.7%)	N = 36 (30.3%)	43.9%	*p* = .012

Note. LGCA = Latent Growth Curve Analyses; HoNOSCA = Health of the National Outcome Scales for Children and Adolescents. The HoNOSCA items can be scored using a five-point Likert scale: 0 “No problem”, 1 “Minor problem requiring no action”, 2 “Mild problem but definitely present”, 3 “Moderately severe problem” and 4 “Severe to very severe problem”. The glossary specifies that a rating of 2 or higher indicates a clinically significant symptom, worthy of clinical attention (“problematic”). A rating of 0 or 1 indicates no clinical problem (Non-problematic). NS = non-significant.

Effects of Youth Flexible Assertive Community Treatment: Outcomes of an 18-Months Observational Study
Social Psychiatry and Psychiatric Epidemiology
Marieke Broersen, Daan H. M. Creemers, Nynke Frieswijk, Ad A. Vermulst, Hans Kroon

Correspondence:
Marieke Broersen
m.broersen@ggzoostbrabant.nl
GGZ Oost Brabant, Oss, the Netherlands
Tranzo – Tilburg School of Social and Behavioral Sciences, Tilburg University, Tilburg, The Netherlands
