# Supplementary material for: Effects of Youth Flexible Assertive Community Treatment: outcomes of an 18-month observational study
Source: Soc Psychiatry Psychiatr Epidemiol. 2023 Jun 6;59(5):745–58. doi: 10.1007/s00127-023-02508-x (PMC11087363; doi:10.1007/s00127-023-02508-x)
Supplement: Supplementary file 2 — Supplementary file2 (PDF 81 KB) [file 127_2023_2508_MOESM2_ESM.pdf]

Supplement 1 Statistical Appendix

In this study, we assumed that the missing data were Missing at Random (MAR). Besides this missing data mechanism, two other mechanisms are known in literature: Missing Completely At Random (MCAR) and Missing Not At Random (MNAR). MCAR means purely haphazard missingness completely unrelated to the data. MAR means that missingness on a variable Y is related to one or more other measured variables but not to values of Y itself, whereas MNAR means that missingness of variable Y is related to values of Y itself [1]. To handle missing values, it is mostly assumed (and often reasonably) that an MCAR- or a MAR mechanism is appropriate and that Full Information Maximum Likelihood estimators or Multiple Imputation are the predominant approaches to handle missing values [2].

However, the missingness in the data of this study was substantial and could not be ignored [2]. To examine the dropout effect on parameter estimates for LGC models, Enders [2] and Coertjens et al. [3] proposed sensitivity analyses. An LGC analysis should be conducted first by assuming MAR. The results should then be compared with LGC analyses under several conditions of MNAR. The validity of the LGC results under MAR would be enhanced if we obtained the same results under MNAR. If the results were inconsistent, the MAR results would have to be interpreted cautiously. This would not mean that either the MAR results are incorrect or that the MNAR results are correct, because several assumptions of MAR and MNAR are untestable [1-2]. We used two well-known MNAR models to study the effect of dropout on the parameters of the growth model: the selection model [4-5] and the pattern mixture model [6]. The process and analysis of these two models are available on request to improve the readability of this study[1]. The sensitivity analyses indicated that the results of the two MNAR models are not fully in line with the results of the MAR model for the client growth models and the mental health worker growth models. Accordingly, the growth curve results in this study must be considered with caution.

Effects of Youth Flexible Assertive Community Treatment: Outcomes of an 18-Months Observational Study

Social Psychiatry and Psychiatric Epidemiology

Marieke Broersen, Daan H. M. Creemers, Nynke Frieswijk, Ad A. Vermulst, Hans Kroon

Correspondence:
Marieke Broersen
m.broersen@ggzoostbrabant.nl
GGZ Oost Brabant, Oss, the Netherlands
Tranzo – Tilburg School of Social and Behavioral Sciences, Tilburg University, Tilburg, The Netherlands

[1] The description of the sensitivity analyses can be requested from the first author.

SUPPLEMENTAL REFERENCES

1. Enders CK (2011) Analyzing Longitudinal Data With Missing Values. Rehabil Psychol 56(4):267-288. doi: 10.1037/a0025579

2. Enders, CK (2011) Missing Not at Random models for Latent Growth Curves. Psychol Methods 16(1):1-16. https://doi.org/10.1037/a0022640

3. Coertjens L. Donche V, De Maeyer S, Vanthournout G, Van Petegem P (2017) To what degree does the missing-data technique influence the estimated growth in learning strategies over time? A tutorial example of sensitivity analysis for longitudinal data. PLoS ONE 12(9): e0182615. https://doi.org/10.1371/journal.pone.0182615

4. Diggle P, Kenward MG (1994) Informative Dropout in Longitudinal Data Analysis. J R Stat Soc Ser C Appl Stat 43(1):49-73. https://doi.org/10.2307/2986113

5. Enders CK (2010) Applied Missing Data Analysis. The Guilford Press, New York.

6. Little RJA (1993) Pattern Mixture Models for Multivariate Incomplete Data. J Am Stat Assoc 88:125-134. DOI: 10.1080/01621459.1993.10594302
